# Profile of tRNA-derived short non-coding RNAs during monocyte differentiation and their role in macrophage survival

**DOI:** 10.1080/15476286.2025.2525886

**Published:** 2025-06-27

**Authors:** Anahita Jayaram, Sanjeev Galande, Soroush Sharbati, Deepak Deshpande, Kamlesh Pawar

**Affiliations:** a Centre of Excellence in Epigenetics (CoEE), Department of Life Sciences, School of Natural Science, Shiv Nadar Institution of Eminence Deemed to be University, Delhi NCR, Greater Noida, India; b Institute of Veterinary Biochemistry, School of Veterinary Medicine, Berlin, Germany, Freie Universität Berlin institution; c Center for Translational Medicine, Sidney Kimmel Medical College, Thomas Jefferson University, Philadelphia, PA, USA

**Keywords:** Monocyte, macrophage, differentiation, ncRNA, tRNA-fragments, apoptosis

## Abstract

Monocytes, a type of leukocytes, are key players in immune responses, transitioning to macrophages at infection sites. Differentiation, which is crucial for macrophage survival and resistance to apoptosis, is a tightly regulated process. Based on our earlier findings implicating the 5'-fragment of tRNA^HisGUG^ (5'-HisGUG) in macrophage biology, we specifically focused on these fragments to investigate its regulation during monocyte-to-macrophage differentiation and its role in macrophage survival. We performed small RNA sequencing during monocyte-to-macrophage differentiation using THP-1 cells and identified 5'-HisGUG as a prominently regulated tDR. Notably, transfection of 5'-HisGUG in macrophages decreased survival under apoptotic stress. We propose that 5'-HisGUG can also act as an obstructive RNA (oRNA), and its reduction is critical for macrophage survival under stress.

## Introduction

Monocytes, a distinct subset of circulating leukocytes, play a vital role in innate immunity as the first line of defence against pathogens [1,2]. Upon stimulation by factors such as macrophage colony-stimulating factor (M-CSF) and interleukin-3 (IL-3), they differentiate into macrophages, including M1 and M2 phenotypes, which is the first critical step in the immune responses to pathogens [1,3]. During monocyte-to-macrophage differentiation, significant changes occur not only in protein expression but also in various RNA species. Transcriptomic analysis has revealed numerous genes involved in this process [4,5], and recent studies have highlighted the critical roles of short non-coding RNAs (sncRNAs), such as miRNAs, and long non-coding RNAs (lncRNAs), in facilitating monocyte to macrophage differentiation [6–8]. Advances in sequencing technologies continue to reveal previously unknown RNAs [9,10], whose functions in biological processes such as monocyte-macrophage differentiation remain to be fully established. Among these, tRNA-derived short non-coding RNAs have gained increasing attention in recent years [11–13]. One of the fragments, called 5'-tRNA halves, is produced through specific cleavage at the anticodon loop by the enzyme angiogenin [14,15] and, is among the most abundant. These fragments are formed with a 2', 3'-cyclic phosphate group at their 3' end and have been shown to regulate cell proliferation, immune responses to infection, and stress granule formation [13–15]. Our recent studies demonstrated that 5'-fragment of tRNA^HisGUG^ (5'-HisGUG) regulate macrophage function (i.e. cytokine production) via TLR7 [16–18]. However, the role of tDRs in monocyte differentiation has not been established. Another unique property of macrophages compared to monocytes is their ability to resist cell death [19]. Macrophages are more resistant to apoptosis due to alterations in pro-apoptotic signalling pathways than their monocytic precursors [20]. However, the molecular players that promote macrophage survival are not well established. In present study, we hypothesized that tDRs species play a critical role in macrophage survival.

In the present study, we explore the expression and possible function of 5'-fragment of tRNA^HisGUG^ (5'-HisGUG) and determined the tDR profile during THP-1 monocytes differentiation into macrophages. Using small-RNA sequencing, we analysed tDR expression in THP-1 monocytes and human monocyte-derived macrophages (HMDMs). Furthermore, we evaluated the effect of transfecting HMDMs with the most significant downregulated 5'-tRNA halves on macrophage survival in response to an apoptotic stimulus. Our findings demonstrate that 5'-fragment of tRNA^HisGUG^ (5'-HisGUG) is the most significantly altered tDR in macrophages. 5'-HisGUG is highly expressed in monocytes and its expression is significantly downregulated in macrophages. Furthermore, the presence of 5'-tRNA^HisGUG^ significantly reduced HMDM survival when exposed to an apoptotic stimulus, indicating its potential role in modulating cell survival promoting macrophage survival.

## Results

### Abundance of tDrs and other small RNAs during THP-1 differentiation to HMDMs

**Figure 1.**
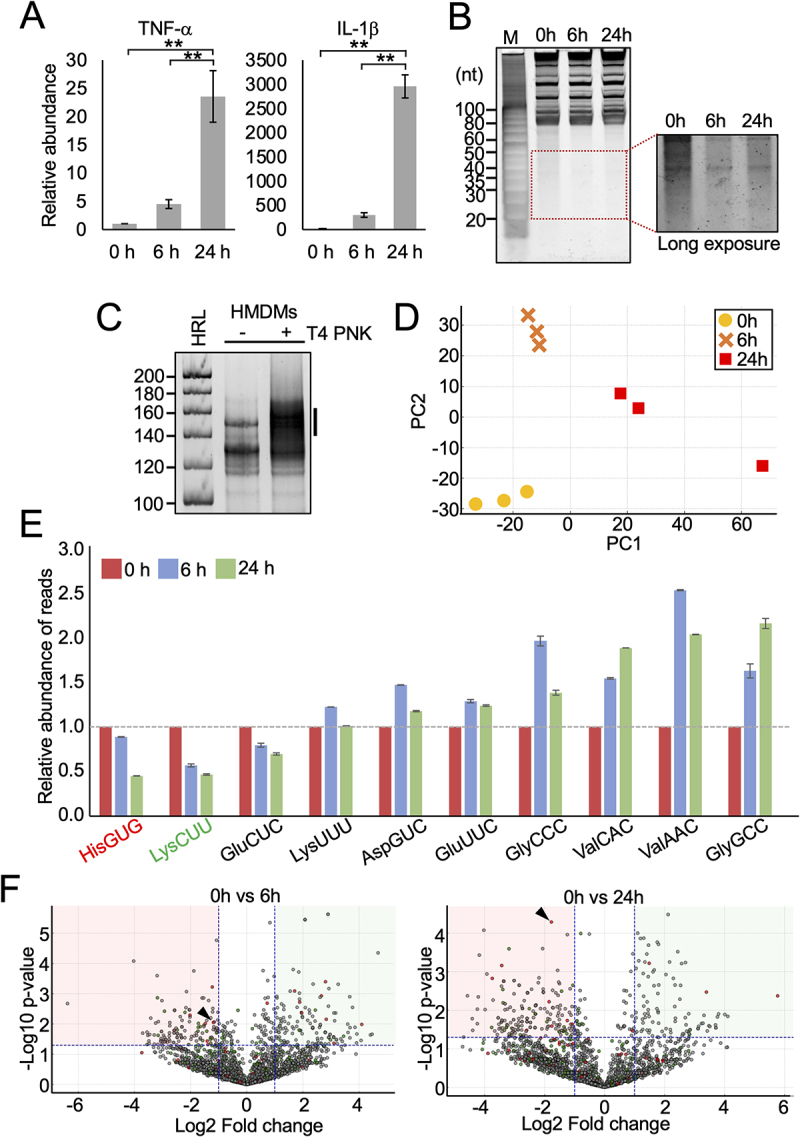
Differential regulation of tDR in THP-1 and PMA induced differentiated HMDMs. (A) THP-1 were incubated with PMA for 0 h, 6 h and 24 h to differentiate monocyte to macrophage. Total RNAs isolated from the cells were subjected to RT-qPCR for the indicated mRNAs. The quantified mRNA levels were normalized to the levels of GAPDH mRNA. (B) Total RNAs from THP-1 and PMA induced HMDMs were visualized after denaturing PAGE by staining with SYBR gold. The short RNA regions (20–50 nt) are shown with a box. (C) Total RNAs from HMDMs were subjected to small RNA-seq, which amplified 138–168-bp cDNA products (5'-adapter, 55 bp; 3'-adapter, 63 bp; and thereby estimated inserted sequences, 20–50 bp). The cDNAs in the region designated with a line were purified and subjected to Illumina sequencing. (D) Principal Component Analysis (PCA) of tRNA expression profiles during differentiation. PCA of tRNA expression in THP-1 cells at 0 h, 6 h, and 24 h post PMA treatment shows clear timepoint-specific clustering, indicating dynamic changes in tRNA fragment profiles during macrophage differentiation. (E) Dynamic expression of top tRNA fragments over time. Relative read abundance of specific tRNA fragments at 0 h, 6 h, and 24 h post PMA treatment shows time-dependent up- or downregulation, indicating dynamic changes during macrophage differentiation. (F) Volcano plots showing time-dependent regulation of tRNA fragments. Modest changes were observed at 6 h post-treatment, while 24 h showed widespread up- and downregulation, indicating a progressive shift in tRNA fragment expression during differentiation. Red, green and grey dots represent RNA fragments derived from tRNA^HisGUG^, tRNA^LyCUU^ and other tRNAs respectively. 5'-HisGUG (G1-G34) is shown with arrowhead.

To induce the differentiation of THP-1 cells into HMDMs, we treated THP-1 with PMA for the indicated period. PMA has been demonstrated as a potent inducer of THP-1 differentiation into HMDMs [21]. PMA treatment activates NF-κB signalling and upregulates of cytokine mRNA expression [22,23]. We assessed the levels of TNFα and IL-1β mRNAs after PMA treatment as a marker of monocyte differentiation into macrophages. As depicted in Figure 1A, there was significant upregulation of both IL-1β and TNF-α in cells exposed to PMA. To visualize the abundance of small RNAs in monocytes and macrophages, total RNA from both cell types was resolved on a urea-PAGE gel and stained with SYBR Gold nucleic acid dye. Staining of total RNAs revealed the presence of small RNAs with ~ 20–50 nucleotides (nt) in length in monocytes (Figure 1B), and PMA treatment significantly reduced the abundance of small RNAs that are expected to include tDR and other small RNAs [16].

To identify the expression profiles of small RNA species, total RNAs of THP-1 cells treated with PMA for 0 h, 6 h and 24 h were sent for small RNA sequencing. We determined the levels of all short RNA species containing cyclic phosphate (cP), phosphate (P) or a hydroxyl group (OH) at the 3' end. For this, total RNAs were first treated with T4 polynucleotide kinase (T4 PNK), which removes cP and P from the 3' end of RNAs, and then subjected to short RNA-seq procedure [16]. The dependency on T4 PNK treatment confirmed that the amplified bands contain RNA with -cP, -P, or -OH at the 3' end (Figure 1C). Illumina sequencing of the cDNA libraries generated roughly 20.3–24.7 million reads corresponding to various small RNAs. Number of mapped reads are shown in Table S1. PCA of tRNA mapped reads showed clear time-dependent clustering of samples, with tight grouping of biological replicates (Figure 1D). 6 h samples separated along PC2, indicating an early shift, while 24 h samples dispersed along PC1, reflecting progressive divergence. When we examined the reads mapped to individual tRNAs, we observed a consistent downward trend for tRNA^HisGUG^, tRNA^LysCUU^, and tRNA^GluCUC^ (Figure 1E), which aligns with the downregulation of small RNAs observed in the urea-PAGE analysis. Volcano plots comparing RNA expression at 6 h and 24 h post-treatment to baseline (0 h) revealed a time-dependent regulatory pattern (Figure 1F). At 6 h, only a modest number of RNA fragments showed significant changes, indicating an early and limited response. In contrast, the 24 h comparison showed a broader distribution of significantly up- and downregulated fragments, reflecting a stronger and more widespread regulatory shift.

### PMA-induced differentiation of THP-1 cells into HMDMs significantly alters the reads mapped to tRNA^HisGUG^

**Figure 2.**
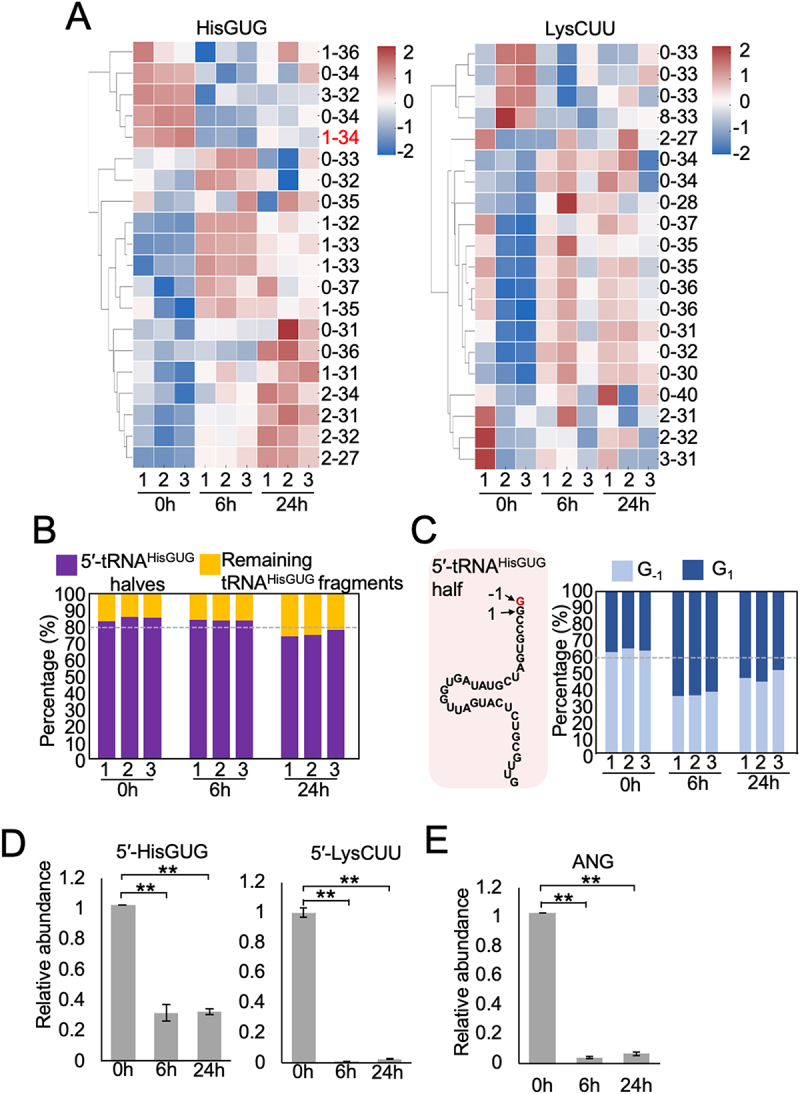
PMA-induced differentiation modulates 5'-tRNA^HisGUG^ fragment abundance and ANG expression in THP-1 cells. (A) Heatmaps showing expression of individual fragments derived from tRNA^HisGUG^ and tRNA^LysCUU^ across 0 h, 6 h, and 24 h timepoints post PMA treatment. Data represent z-scores of normalized read counts; each column represents a biological replicate (*n* = 3). (B) Proportional distribution of 5'-tRNA^HisGUG^ halves versus other fragments mapped to tRNA^HisGUG^. The 5'-halves (purple) dominate the mapped reads at all timepoints, although their relative proportion decreases at 24 h. (C) Base-specific analysis of 5'-tRNA^HisGUG^ half isoforms categorized by the first nucleotide (G_−1_ vs. G_1_). The 5'-tRNA^HisGUG^ fragments with the first nucleotide G_−1_ is labelled as position 0 in Figure 1A. (D) Total RNAs from THP-1 as well as PMA induced differentiated HMDMs were subjected to TaqMan RT-qPCR for 5'-HisGUG and 5'-LysCUU. The quantified RNA levels were normalized to the levels of 5S rRNA. (E) Total RNAs isolated from the cells were subjected to RT-qPCR for the *ANG* mRNAs.

Heatmap analysis of the top 20 tRNA-derived fragments from tRNA^HisGUG^ and tRNA^LysCUU^ revealed distinct expression patterns over time (Figure 2A). Top abundant tRNA^HisGUG^-derived fragments showed consistent downregulation at 24 hours, with clear clustering across replicates, indicating coordinated, time-dependent regulation during PMA-induced differentiation. In contrast, tRNA^LysCUU^-derived fragments exhibited more heterogeneous expression with no consistent directional shift, indicating greater variability and potentially weaker association with the differentiation process. These results support the selective regulation and possible functional relevance of tRNA^HisGUG^-derived fragments in macrophage differentiation. The sequence information of top 20 tRNA-derived fragments from tRNA^HisGUG^ and tRNA^LysCUU^ are provided in Table S2. Among tRNA^HisGUG^-derived fragments, 5'-tRNA^HisGUG^ halves consistently represented over 80% across all timepoints, with a slight decrease at 24 h (Figure 2B). This suggests they are the predominant and likely functionally relevant species during differentiation. Analysis of 5'-tRNA^HisGUG^ variants revealed a shift from the G-₁-starting fragment to the canonical G₁ fragment over time (Figure 2C). While the G-₁ variant was more abundant at 0 h, the G₁ variant increased at 6 h and 24 h, suggesting a regulated change in tRNA cleavage specificity during differentiation. The commonly expressed and regulated 5' fragment that is 5'-HisGUG [16,17], were analysed to determine their levels in monocytes and macrophages. TaqMan RT-qPCR was performed to quantify the changes in the abundance of 5'-HisGUG. As shown in Figure 2D, significant downregulation of 5'-HisGUG and 5'-LysCUU was observed during PMA-induced differentiation further validating our unbiased small RNA sequencing studies described in Figure 1.

In mammalian cells, angiogenin (ANG) cleaves the anticodon loops of tRNAs to produce tRNA halves [15,24]. To explore whether ANG may be involved in tRNA half production during differentiation, we quantified ANG mRNA levels in THP-1 cells and HMDMs. As shown in Figure 2E, ANG transcript levels significantly decreased at 6 h and 24 h post PMA treatment.

### The presence of 5'-HisGUG decreases the cell survival in response to apoptotic stimuli

**Figure 3.**
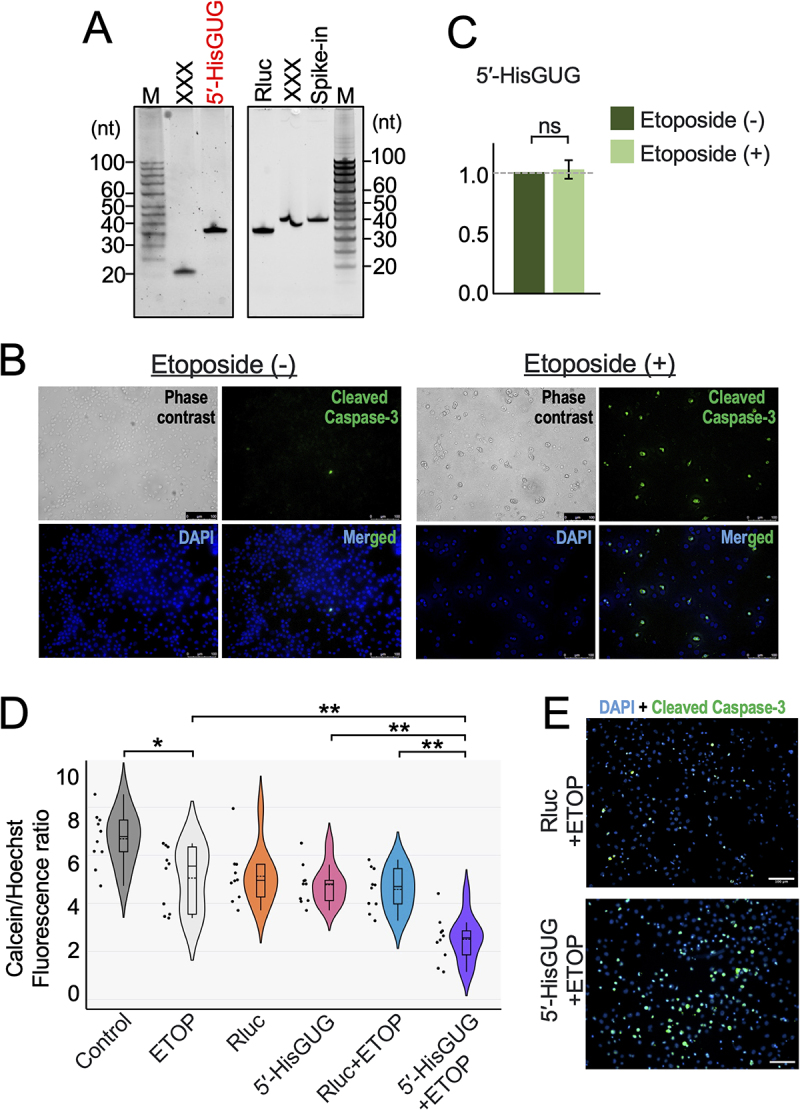
The presence of 5'-HisGUG affects macrophage cell survival when exposed to apoptotic stimuli. (A) Indicated synthetic RNAs were synthesized by in vitro transcription, gel-purified, and analysed with denaturing PAGE. Irrelevant RNAs to the current study were marked with XXX. (B) Induction of apoptosis by etoposide was tracked by detecting cleaved caspase-3 (green). Nuclei were counterstained with DAPI (blue). Scale bars indicate 100 μm. (C) Total RNAs from HMDMs treated with etoposide were subjected to TaqMan RT-qPCR for 5'-HisGUG. The quantified RNA levels were normalized to the levels of 5S rRNA. (D) HMDMs were treated with etoposide and/or transfected with RNA as indicated. Cell survival was analysed using the Calcein fluorometric assay. (E) HMDMs were first transfected with the indicated RNAs and apoptosis was induced by etoposide. Apoptosis induction was tracked by detecting cleaved caspase-3 (green) and nuclei were counterstained with DAPI (blue). Scale bars indicate 100 μm.

Upon differentiation from monocytes, macrophages acquire resistance to apoptosis [20]. Since 5'-HisGUG (1–34 nt) was the most significantly downregulated tRNA derived fragment we tested whether 5'-HisGUG is linked to the induction of apoptosis. We transfected *in vitro* synthesized (Figure 3A) 5'-HisGUG or Rluc (transfection control [25]) into PMA-differentiated macrophages and then induced apoptosis using etoposide [26]. Apoptosis in HMDMs was confirmed by cleaved caspase 3, with etoposide treatment inducing this response (Figure 3B). To understand if 5'-HisGUG is expressed in response to etoposide induced apoptosis in HMDMs, we performed TaqMan-based qPCR to measure 5'-HisGUG levels in macrophages following etoposide treatment. As shown in Figure 3C, we observed no significant change in endogenous 5'-HisGUG expression across multiple replicates. We then assessed cell survival after transfection using the Calcein fluorometric assay. As shown in Figure 3D, 5'-HisGUG transfected cells exhibited reduced cell survival compared to control RNA transfected cells after induction of apoptosis by etoposide. Transfection with 5'-HisGUG before etoposide treatment upregulated cleaved caspase 3 levels in HMDMs (Figure 3E). Etoposide-treated macrophages with lower fluorometric readings compared to untreated cells were used as an assay control. Our earlier study confirmed that *in vitro* synthesized RNA does not activate cytoplasmic RIG-I [16].

## Discussion

Based on our earlier studies demonstrating that 5'-fragment of tRNA^HisGUG^ (5'-HisGUG) functions as a Toll-like receptor 7 (TLR7) ligand and regulates macrophage inflammatory responses [16,18], we hypothesized that this fragment may play a broader regulatory role during monocyte-to-macrophage differentiation and survival. To identify the regulating tRNA-derived fragment (tDRs), small RNA sequencing was performed. Sequencing data revealed that fragments derived from tRNA^HisGUG^ were the most significantly reduced during this transition, with the 5'-HisGUG (G1–G34) fragment showing the significant downregulation at 24 hours post PMA treatment. Although multiple isoforms of 5'-HisGUG were detected with some variability across biological replicates, the G1–G34 isoform consistently emerged as the most abundant and prominently regulated fragment, especially at the 24-hour time point. This consistent enrichment, combined with strong prior functional evidence from our previous studies identifying 5'-HisGUG as a TLR7 ligand and a regulator of macrophage biology [16,18], provided the rationale for selecting this fragment for further mechanistic investigation. We also observed downregulation of fragments derived from tRNA^LysCUU^ and tRNA^GluCUC^. For 5'-LysCUU, both small RNA sequencing and qPCR consistently detected its downregulation, although the degree of change was more pronounced in qPCR. This variation likely reflects differences in platform sensitivity and quantification, with qPCR providing higher sensitivity for specific fragments, while sequencing offers a broader but less quantitative assessment. Despite these differences, both methods consistently confirmed the downregulation of 5'-LysCUU during differentiation.

Since macrophages are more resistant to apoptosis, the significant reduction in 5'-HisGUG production suggests a potential regulatory role in this process. Although 5'-HisGUG was identified as a highly regulated tDR during monocyte-to-macrophage differentiation, our study focused on its role in macrophage survival. Future investigations will be necessary to directly assess whether 5'-HisGUG also modulates the differentiation process itself. Moreover, fragments from other RNAs, such as rRNA, may also contribute to macrophage differentiation or survival, functioning either in coordination with tRNA-derived fragments or independently and warrant further investigation. In this study, by focusing on the 5'-HisGUG fragments, we propose that it may function as an obstructive RNA (oRNA), and that reducing 5'-HisGUG levels is pivotal for macrophage viability under stress conditions.

The THP-1 cell line, derived from human leukaemia monocytes, served as our model for studying monocyte-to-macrophage differentiation [27–29]. PMA-induced differentiation [21,30] activates protein kinase C (PKC) [31,32], triggering pathways that upregulate transcription factors such as NF-κB and c-Jun [33,34], which are essential for macrophage functions [35]. Through phospho-RNA sequencing, we characterized tDR expression profiles in THP-1 monocytes and PMA-induced HMDMs. Our results showed that specific tRNAs contribute to tDR production, with substrates such as tRNA^HisGUG^, tRNA^AspGUC^, tRNA^GluCUC^, tRNA^GlyGCC^, and tRNA^LysCUU^ dominating in PMA-induced HMDMs. In previous studies, LPS treatment induced tDR production from tRNA^HisGUG^, tRNA^ValCAC^ and tRNA^ValAAC^ [16,17] in HMDMs. Our study suggests that PMA, similar to LPS, modulates the tDR expression profile in monocytes/macrophages. Among all, 5'-HisGUG (G1-G34) was the most significantly downregulated tDR. The low levels of 5'-HisGUG in macrophages may result from their selectively packaging and secretion via extracellular vesicles (EVs) without compensatory intracellular accumulation, as macrophages are known to release such vesicles [36,37]. In fact, a previous study by our group demonstrated the selective packaging and secretion of 5'-HisGUG by macrophages, supporting this hypothesis [16]. In cancer cells, 5'-HisGUG has been linked to cellular proliferation [38], and its inhibition reduces tumour growth [39]. The higher 5'-HisGUG expression in THP-1 monocytes may support their proliferation, whereas lower levels in HMDMs could correspond to the loss of cell division.

PMA-induced differentiation of THP-1 cells reduced the expression of 5'-tRNA fragments, possibly due to decreased Angiogenin (ANG) levels. ANG cleaves the anticodon loop of specific tRNAs to generate tRNA halves [15,24]. While we acknowledge that mRNA levels do not always directly reflect protein abundance or enzymatic activity, the observed downregulation of ANG mRNA suggests a potential reduction in ANG-mediated cleavage of tRNAs during macrophage differentiation. The selective susceptibility of tRNAs to cleavage remains unclear, but modifications to tRNA may contribute to these biases. While LPS upregulates ANG expression [16], PMA exposure downregulates ANG expression. These divergent effects highlight the context-dependent roles of NF-κB, other signalling molecules, and transcription factors [40]. Further studies are necessary to elucidate the upstream mechanisms involved in ANG expression regulation

Monocytes are prone to apoptosis, whereas macrophages are resistant to apoptosis during disease progression [20,41]. We observed a significant reduction in 5'-HisGUG levels in macrophages compared to that in monocytes. While the downregulation of 5'-HisGUG during PMA-induced differentiation suggested a potential role in either the differentiation process or apoptosis resistance, we focused on the latter due to prior studies linking tDRs with Cyt c-related apoptotic pathways [42,43]. Our previous findings showing that 5'-HisGUG activates TLR7 and modulates inflammatory gene expression further support its broad involvement in macrophage biology [16,18]. However, we acknowledge that 5'-HisGUG or other small RNAs could also play a direct role in the differentiation process. To investigate the role of 5'-HisGUG in apoptosis, we transfected HMDMs with 5'-HisGUG. While it did not trigger apoptosis on its own, the presence of 5'-HisGUG significantly increased cell susceptibility to apoptosis upon etoposide treatment. This suggests that 5'-HisGUG acts not as a direct inducer but as a sensitizer or modulator of apoptotic pathways. This also rules out the possibility that the observed effect is simply due to the use of high amounts of 5'-HisGUG during transfection. While the transfection dose was based on our previous method [16], future studies quantifying endogenous 5'-HisGUG and adjusting transfection levels accordingly would enhance both mechanistic understanding and translational relevance.

Next-generation sequencing of the cytochrome c-ribonucleoprotein (Cyt c-RNP) complex revealed 20 enriched tRNA-derived fragments (tDRs), but 5'-HisGUG was absent [44]. Instead, a 3'-HisAUG fragment was detected, suggesting that 5'-HisGUG does not directly bind Cyt c under the tested conditions. This discrepancy may stem from contextual differences – osmotic stress in previous work versus monocyte-to-macrophage differentiation in our study. Functionally, 5'-HisGUG alone did not induce apoptosis in macrophages. However, when followed by etoposide treatment, it significantly enhanced apoptotic response compared to control RNA. These findings suggest that 5'-HisGUG acts not as a pro-apoptotic signal itself but as a sensitizer to apoptotic stress.

We propose that 5'-HisGUG may function as an obstructive RNA (oRNA), interfering with the binding or function of other cytoprotective tDRs. By displacing protective RNAs from key components such as Cyt c or Apaf-1 [44–46], it may lower the apoptotic threshold. This model underscores the nuanced and context-dependent roles of tDRs in regulating cell fate.

In summary, our study identifies 5'-HisGUG as a key regulated tDR during monocyte-to-macrophage differentiation and suggests it functions as an oRNA modulating apoptosis and macrophage survival. These findings advance our understanding of tDRs in immune regulation and cell fate control.

## Experimental procedures

### Cell culture, PMA treatment and apoptosis induction by etoposide

THP-1 human acute monocytic leukaemia cells (American Type Culture Collection, Manassas, Virginia, USA) were cultured in RPMI 1640 medium (HiMedia, India) supplemented with 10% FBS (HiMedia, India) and penicillin-streptomycin (Thermo Fisher Scientific, USA). THP-1 cells were differentiated into human monocyte-derived macrophages (HMDMs) using 10 nM phorbol 12-myristate 13-acetate (PMA; Sigma-Aldrich, St. Louis, MO, USA), as described previously [16,47,48]. Cells were incubated with PMA for 0, 6, or 24 hours at 37°C in a CO₂ incubator. Cells treated for 24 hours were considered fully differentiated HMDMs. To induce apoptosis in HMDMs, cells were incubated in presence of 25 µM Etoposide (MP Biomedicals) for 16 h [26,49].

### Immunofluorescence microscopy

The successful induction of apoptosis in HMDMs was confirmed by detecting the presence of cleaved caspase 3 using immunofluorescence microscopy as described previously [48]. In brief, after 16 h of incubation in the presence of etoposide, the cells were washed with PBS and fixed in 4% Roti-Histofix (Carl Roth). Immunostaining was performed using a 1:300 dilution of the cleaved caspase 3 (Asp175) antibody (#9661, CST) followed by incubation with goat anti-rabbit IgG (H+L) Cross-Adsorbed Secondary Antibody, Alexa Fluor 488 (Thermo Scientific) at 1:300 dilution. Nuclei were counterstained using DAPI (Sigma-Aldrich). Microscopic photographs were obtained using an inverted fluorescence microscope (DMI6000 B, Leica).

### RNA isolation, TaqMan RT-qPCR and RT-qPCR

Following PMA treatment, total RNA was isolated from cells using RNAiso Plus (Takara Bio, Kusatsu, Shiga, Japan). TaqMan RT-qPCR was performed for the quantification of 5'-tRNA halves as previously described [16]. In short, total RNA was treated with T4 Polynucleotide Kinase (T4 PNK) to replace the cP group with a hydroxyl group, and then ligated to a 3'-RNA adaptor using T4 RNA ligase. TaqMan qPCR was then performed on the ligated RNA using the One Step PrimeScript RT-PCR Kit (Takara Bio, Kusatsu, Shiga, Japan), forward and reverse primers and 200 nM of specific TaqMan probes complimentary to the boundary of the target RNA and 3'-adaptor sequence (sequence information is shown in [17]). Data was normalized using U6 ribosomal RNA (rRNA) or 5S rRNA.

Standard RT-qPCR was performed to quantify mRNA levels of cytokines IL-1β and TNF-α and was normalized using GAPDH and RPLP0 housekeeping genes as described previously [16]. In brief, total RNA was first treated with DNase I (Promega, Madison, Wisconsin, USA), and then used as a template to prepare cDNA by reverse transcription using RevertAid Reverse Transcriptase (Thermo Fisher Scientific, USA) and a reverse primer. The prepared cDNA was then amplified using SYBR Green PCR Master Mix (Thermo Fisher Scientific, USA) with forward and reverse primers specific for the target genes (sequence information is shown in [17] and Table S3).

### Small RNA-seq and bioinformatics

Total RNA from PMA-treated and untreated cells was treated with T4 PNK and subjected to small RNA sequencing. The amplified cDNAs were gel purified and were sequenced using the Illumina NovaSeq 6000 system. The reads were analysed by a previously described bioinformatic pipeline and were mapped to tRNAs obtained from GtRNAdb, and then to mature rRNAs as described [16].

### In vitro RNA synthesis

*In vitro* transcription was employed to prepare synthetic 5'-tRNA halves according to a method described previously [17]. The dsDNA templates were synthesized using T4 DNA Polymerase (New England Biolabs, UK), and overlapping forward and reverse primers. Native PAGE was performed using 40% 19:1 acrylamide, N,N,N',N' -Tetramethylethylenediamine (TEMED) and Ammonium Persulphate (APS) to confirm the success of the reaction. The templates were subjected to *in vitro* transcription using T7 RNA Polymerase in a reaction carried out at 37°C for 10 hours. Synthetic RNAs were resolved using denaturing PAGE with single-nucleotide resolution. This was prepared using 12% 7 M urea solution, TEMED and APS, and run using 1X Tris-Borate-EDTA (TBE) buffer at 200 V. The gel was stained with SYBRGold visualizing dye (Thermo Fisher Scientific). Gel-extracted RNA was purified using a gel extraction buffer and filtered. After purifying the RNA and subjecting it to Urea PAGE separation, a distinct RNA band was visualized using Ethidium bromide (Bio-Rad, USA) staining. Primer sequence and synthesized RNA sequences are given in Table S3 and Table S4 respectively.

### Transfection of macrophages and Calcein fluorometric assay

THP-1 cells (1 × 10^6^) were differentiated into HMDMs, as described above. The HMDMs then transfected using RNAiMAX (Thermo Fisher Scientific) with 50 pmol 5'-HisGUG or control RNA as described previously [16,25], a dose chosen to elicit biological responses without causing non-specific cytotoxicity or activating unrelated RNA sensors.

After 24 h of transfection, cells were incubated in the presence or absence of Etoposide for 16 h [26]. Following treatments, the viability of HMDMs at 16 h was evaluated using Calcein-AM/Hoechst staining [50]. This assay measures the number of viable cells using the Calcein signal, while the total cell count was determined by Hoechst staining. Cell survival after induction of apoptosis was evaluated by calculating the ratio of viable cells to total cells and comparing it to that of untreated cells.

### Statistical analysis

Statistical significance between samples was determined using a two-tailed t-test. Differential expression analysis was performed using *PyDESeq2* in Python (Python 3.12.6). Log₂ fold changes and adjusted *p-*values were used to identify significant RNAs. Volcano plots were generated with *matplotlib*, PCA with *scikit-learn* on variance-stabilized data, and hierarchical heatmaps using z-score normalized values with *seaborn.clustermap*.

## Supplementary Material

Supplementary_Table.docx

## Data Availability

The Next-Generation Sequence libraries are publicly available from the NCBI Sequence Read Archive (accession No. SRR33452038, SRR33452039, SRR33452040, SRR33452041, SRR33452042, SRR33452043, SRR33452044, SRR33452045, SRR33452046). Any underlying data with respect to the manuscript will be made available upon reasonable request.
