# Supplementary material for: Profile of tRNA-derived short non-coding RNAs during monocyte differentiation and their role in macrophage survival
Source: RNA Biol. 2025 Jun 27;22(1):1–9. doi: 10.1080/15476286.2025.2525886 (PMC12233707; doi:10.1080/15476286.2025.2525886)
Supplement: Supplementary_Table.docx [file KRNB_A_2525886_SM4470.docx]

Table S1: Read numbers of sequence libraries

| Sample name | Raw reads | Reads after adaptor cut | Reads after adaptor cut and size selection | tRNA | rRNA | Other |
| --- | --- | --- | --- | --- | --- | --- |
| 0H_1 | 20,382,319 | 20,267,884 | 7,149,630 | 261,661 | 5,437,042 | 1,450,927 |
| 0H_2 | 23,358,485 | 23,257,522 | 9,369,255 | 374,726 | 6,973,302 | 2,021,227 |
| 0H_3 | 22,828,785 | 22,730,548 | 9,090,240 | 343,056 | 6791739 | 1955445 |
| 6H_1 | 23,822,750 | 23,700,233 | 8,333,588 | 311,625 | 6056242 | 1965721 |
| 6H_2 | 24,751,768 | 24,647,268 | 8,243,177 | 308,244 | 5992611 | 1942322 |
| 6H_3 | 22,393,729 | 22,289,491 | 8,062,129 | 311,211 | 5833056 | 1917862 |
| 24H_1 | 23,445,395 | 23,334,664 | 9,700,563 | 298,258 | 7074405 | 2327900 |
| 24H_2 | 24,190,235 | 24,076,526 | 9,899,365 | 303,217 | 7173554 | 2422594 |
| 24H_3 | 21,370,426 | 21,271,088 | 8,899,155 | 243,201 | 6641827 | 2014127 |

Table S2: Sequence information of tRNA derived RNA

| tRNA | Sequence | Nucleotide position | Length |
| --- | --- | --- | --- |
| HisGUG | GCCGTGATCGTATAGTGGTTAGTACTCTGCGTTGT | 1-36 | 35 |
| HisGUG | GGCCATGATCGTATAGTGGTTAGTACTCTGCGCT | 0-34 | 34 |
| HisGUG | CGTGATCGTATAGTGGTTAGTACTCTGCG | 3-32 | 29 |
| HisGUG | GGCCGTGATCGTATAGTGGTTAGTACTCTGCGTT | 0-34 | 34 |
| HisGUG | GCCGTGATCGTATAGTGGTTAGTACTCTGCGTT | 1-34 | 33 |
| HisGUG | GGCCGTGATCGTATAGTGGTTAGTACTCTGCGT | 0-33 | 33 |
| HisGUG | GGCCGTGATCGTATAGTGGTTAGTACTCTGCG | 0-32 | 32 |
| HisGUG | GGCCGTGATCGTATAGTGGTTAGTACTCTGCGTTG | 0-35 | 35 |
| HisGUG | GCCGTGATCGTATAGTGGTTAGTACTCTGCG | 1-32 | 31 |
| HisGUG | GCCGTGATCGTATAGTGGTTAGTACTCTGCGT | 1-33 | 32 |
| HisGUG | GCCATGATCGTATAGTGGTTAGTACTCTGCGC | 1-33 | 32 |
| HisGUG | GGCCGTGATCGTATAGTGGTTAGTACTCTGCGTTGTG | 0-37 | 37 |
| HisGUG | GCCGTGATCGTATAGTGGTTAGTACTCTGCGTTG | 1-35 | 34 |
| HisGUG | GGCCGTGATCGTATAGTGGTTAGTACTCTGC | 0-31 | 31 |
| HisGUG | GGCCGTGATCGTATAGTGGTTAGTACTCTGCGTTGT | 0-36 | 36 |
| HisGUG | GCCGTGATCGTATAGTGGTTAGTACTCTGC | 1-31 | 30 |
| HisGUG | CCGTGATCGTATAGTGGTTAGTACTCTGCGTT | 2-34 | 32 |
| HisGUG | CCGTGATCGTATAGTGGTTAGTACTCTGC | 2-31 | 29 |
| HisGUG | CCGTGATCGTATAGTGGTTAGTACTCTGCG | 2-32 | 30 |
| HisGUG | CCGTGATCGTATAGTGGTTAGTACT | 2-27 | 25 |
| LysCUU | GCCCGGCTAGCTCAGTCGGTAGAGCATGAGACT | 0-33 | 33 |
| LysCUU | GCCCGGCTAGCTCAGTCGGTAGAGCATGAGACC | 0-33 | 33 |
| LysCUU | GCCCGGCTAGCTCAGTCGGTAGAGCATGGGACT | 0-33 | 33 |
| LysCUU | AGCTCAGTCGGTAGAGCATGGGACT | 8-33 | 25 |
| LysCUU | CCGGCTAGCTCAGTCGGTAGAGCAT | 2-27 | 25 |
| LysCUU | GCCCGGCTAGCTCAGTCGGTAGAGCATGAGACTC | 0-34 | 34 |
| LysCUU | GCCCGGCTAGCTCAGTCGGTAGAGCATGGGACTC | 0-34 | 34 |
| LysCUU | GCCCGGCTAGCTCAGTCGGTAGAGCATG | 0-28 | 28 |
| LysCUU | GCCCGGCTAGCTCAGTCGTAGAGCATGAGACTCTTA | 0-37 | 36 |
| LysCUU | GCCCGGCTAGCTCAGTCGGTAGAGCATGAGACTCT | 0-35 | 35 |
| LysCUU | GCCCGGCTAGCTCAGTCGGTAGAGCATGGGACTCT | 0-35 | 35 |
| LysCUU | GCCCGGCTAGCTCAGTCGGTAGAGCATGAGACTCTT | 0-36 | 36 |
| LysCUU | GCCCGGCTAGCTCAGTCGGTAGAGCATGGGACTCTT | 0-36 | 36 |
| LysCUU | GCCCGGCTAGCTCAGTCGGTAGAGCATGAGA | 0-31 | 31 |
| LysCUU | GCCCGGCTAGCTCAGTCGGTAGAGCATGAGAC | 0-32 | 32 |
| LysCUU | GCCCGGCTAGCTCAGTCGGTAGAGCATGAG | 0-30 | 30 |
| LysCUU | GCCCGGCTAGCTCAGTCGGTAGAGCATGAGACTCTTAATC | 0-40 | 40 |
| LysCUU | CCGGCTAGCTCAGTCGGTAGAGCATGAGA | 2-31 | 29 |
| LysCUU | CCGGCTAGCTCAGTCGGTAGAGCATGAGAC | 2-32 | 30 |
| LysCUU | CGGCTAGCTCAGTCGGTAGAGCATGGGA | 3-31 | 28 |

Table S3: Sequence information of primers used in TaqMan qPCR, SYBR green qPCR and invitro RNA synthesis.

| Name | Type | Sequence |
| --- | --- | --- |
| 3′Adaptor | Adaptor (RNA) | /5Phos/GAACACUGCGUUUGCUGGCUUUGAGAGUUCUACAGUCCGACGAUC/3ddC/ |
| 5'-HisGUG-Taq | TaqMan probe | 5HEX/TAGTACTCT/ZEN/GCGTTGAACACTGCGTTTGC/3IABkFQ/ |
| 5'-HisGUG-Fw | Forward | GCTCGCCGTGATCGTATAGT |
| Adaptor-Rev | Reverse | GATCGTCGGACTGTAGAACTC |
| ANG-Fw | Forward | AGAAGCGGGTGAGAAACAAAAC |
| ANG-Rev | Reverse | AGTGCTGGGTCAGGAAGTGTG |
| TNF-𝞪-Fw | Forward | GAGCACTGAAAGCATGATCC |
| TNF-𝞪-Rev | Reverse | CGAGAAGATGATCTGACTGCC |
| IL-1𝞫-Fw | Forward | CAGGCTGCTCTGGGATTCTC |
| IL-1𝞫-Fw | Reverse | CCTGGAAGGAGCACTTCATCT |
| GAPDH-Fw | Forward | GTCTTCACCACCATGGAGAAGG |
| GAPDH-Rev | Reverse | ATGATCTTGAGGCTGTTGTCAT |
| InV-5'-HisGUG-Fw | Forward | GCTTAATACGACTCACTATAGCCGTGATCGTATAG |
| InV-5'-HisGUG-Rev | Reverse | mAmACGCAGAGTACTAACCACTATACGATCACGGC |
| InV-Rluc-Fw | Forward | GCTTAATACGACTCACTATAGGGAGGCAAGCCCGA |
| InV-Rluc-Rev | Reverse | GCGGACAATCTGGACGACGTCGGGCTTGCCTCCC |
| InV-Spike-in-Fw | Forward | GCTTAATACGACTCACTATAGGGAGGTGGGCCAGA |
| InV-Spike-in-Rev | Reverse | CTTGAACAATCTAATGTTTACATCTGGCCCACCTCCC |

Table S4: Sequence information of synthetic RNAs used in the study

| RNA | Sequence | Length |
| --- | --- | --- |
| 5′-HisGUG | GCCGUGAUCGUAUAGUGGUUAGUACUCUGCGUU | 33 |
| Rluc | GGGAGGCAAGCCCGACGUCGUCCAGAUUGUCCGC | 34 |
| Spike-In | GGGAGGUGGGCCAGAUGUAAACAUUAGAUUGUUCAAG | 37 |
